# Speech Movement Variability in People Who Stutter: A Vocal Tract Magnetic Resonance Imaging Study

**DOI:** 10.1044/2021_JSLHR-20-00507

**Published:** 2021-06-22

**Authors:** Charlotte E. E. Wiltshire, Mark Chiew, Jennifer Chesters, Máiréad P. Healy, Kate E. Watkins

**Affiliations:** a Wellcome Centre for Integrative Neuroimaging, Department of Experimental Psychology, Radcliffe Observatory Quarter, University of Oxford, United Kingdom; b Wellcome Centre for Integrative Neuroimaging, Nuffield Department of Clinical Neurosciences, University of Oxford, United Kingdom

## Abstract

**Purpose:**

People who stutter (PWS) have more unstable speech motor systems than people who are typically fluent (PWTF). Here, we used real-time magnetic resonance imaging (MRI) of the vocal tract to assess variability and duration of movements of different articulators in PWS and PWTF during fluent speech production.

**Method:**

The vocal tracts of 28 adults with moderate to severe stuttering and 20 PWTF were scanned using MRI while repeating simple and complex pseudowords. Midsagittal images of the vocal tract from lips to larynx were reconstructed at 33.3 frames per second. For each participant, we measured the variability and duration of movements across multiple repetitions of the pseudowords in three selected articulators: the lips, tongue body, and velum.

**Results:**

PWS showed significantly greater speech movement variability than PWTF during fluent repetitions of pseudowords. The group difference was most evident for measurements of lip aperture using these stimuli, as reported previously, but here, we report that movements of the tongue body and velum were also affected during the same utterances. Variability was not affected by phonological complexity. Speech movement variability was unrelated to stuttering severity within the PWS group. PWS also showed longer speech movement durations relative to PWTF for fluent repetitions of multisyllabic pseudowords, and this group difference was even more evident as complexity increased.

**Conclusions:**

Using real-time MRI of the vocal tract, we found that PWS produced more variable movements than PWTF even during fluent productions of simple pseudowords. PWS also took longer to produce multisyllabic words relative to PWTF, particularly when words were more complex. This indicates general, trait-level differences in the control of the articulators between PWS and PWTF.

**Supplemental Material:**

https://doi.org/10.23641/asha.14782092

Several studies indicate that movements of articulators differ in people who stutter (PWS) compared with people who are typically fluent (PWTF; Frisch et al., 2016; Howell et al., 2009; Jackson et al., 2016; Loucks & De Nil, 2006; Loucks et al., 2007; Sasisekaran, 2013; Smith et al., 2010). These kinematic differences were evident even when the speech produced was perceptually fluent, that is, it appeared to lack disfluencies. The findings indicate that there are general (trait-level) differences in speech motor control in PWS that go beyond the expected movement differences accompanying overt stuttered moments (state level).

Previous speech movement studies in PWS have mostly focused on the measurement of speech movement variability, the amplitude and duration of speech movements, and the muscular effort involved in speech production (reviewed in Wiltshire, 2019). The most consistent finding across these studies was that PWS have greater variability in speech movements across repeated utterances (Smith et al., 1995) when producing targeted jaw movements (Loucks & De Nil, 2006, 2012; Loucks et al., 2007), vowel sounds (Frisch et al., 2016), simple (Sasisekaran, 2013) and complex pseudowords (Smith et al., 2010), and simple sentences (Howell et al., 2009; Jackson et al., 2016; MacPherson & Smith, 2013). In contrast, there is little consensus among studies investigating whether PWS differ in the amplitude (Walsh et al., 2015; Van Lieshout et al., 2014; cf. Namasivayam & van Lieshout, 2008) and duration of movements (McClean & Tasko, 2004; Smith et al., 2010, 2012; Tasko et al., 2007; Usler et al., 2017) or in the movement effort during speech production (Choo et al., 2010; De Andrade et al., 2008; de Felício et al., 2007; Walsh & Smith, 2013).

In this report, we focused on variability in speech movement production since this was the most reliable difference reported in previous studies with PWS. Variability represents a general measure of speech motor control, in which random noise is inserted into the motor plan at some stage prior to execution. It is thought that this noise comes from altered communication of neural signals involved in the transition from planning to the execution of speech. Evidence in support of this comes from reduced structural and functional connectivity between sensory and motor regions of the brain in PWS compared with PWTF (Connally et al., 2014; Neef et al., 2011, 2015; Watkins, 2011; Watkins et al., 2008). However, it is clear that measures of kinematic variability cannot inform us as to whether specific processes within the nervous system are the source of this noise, which may also be linked to cognitive or social factors. Variability, as measured here, can tell us about general differences in the control of speech movements between PWS and PWTF but cannot reveal the source of such variability.

Despite uncertainty about the cause of variability, differences in variability between PWS and PWTF have important theoretical and clinical consequences. Hypotheses that predict differences in the feedforward and feedback control of speech (Bohland et al., 2010; Guenther, 2016; Max et al., 2004) propose that stuttering is caused by a discrepancy between the expected utterance (sensory and auditory predictions) and the actual utterance produced. An error signal may be produced in two ways; either the predictive space is typical but the movements fall outside of this range (as shown by more variable movements), or the movements are typical (as shown by no difference in the variability of movements) but the prediction space is smaller, resulting in less tolerance of movement variability.

The task demands (including complexity or speed of production) can increase the amount of noise in the system. For example, lip aperture movements were recorded using infrared light-emitting diodes in a group of PWS and PWTF as they repeated pseudowords that increased in length (from one to four syllables) and phonological complexity (Smith et al., 2010). Results showed an interaction between group and stimuli, such that variability increased to a greater degree in PWS than in PWTF as the utterances got longer or were more complex (Smith et al., 2010). Similar effects have been found when complexity is modulated by increasing the speed of productions (Namasivayam & van Lieshout, 2008).

The effect of complexity is in keeping with the fact that complex utterances are more likely to result in stuttered moments compared with simple utterances in children (Gaines et al., 1991; Richels et al., 2010) and adults who stutter (Robb et al., 2009). In accordance, speech therapies for stuttering often focus on slowing down speech. Theories of stuttering that propose that PWS have impairment integrating auditory and somatosensory feedback with ongoing motor control predict that producing larger, slower movements would generate more sensory feedback, which could, in turn, be used to gain better control over speech movements. Conversely, smaller, quicker movements would reduce the amount of feedback available, resulting in poorer integration of sensory–motor signals and poorer speech motor control (Namasivayam & Van Lieshout, 2011). The causal direction of the relationship between movement size and speech motor control is unknown. It could be that PWS have weaker speech motor control because they make smaller, shorter movements, thus generating insufficient feedback. Alternatively, PWS could make larger, slower movements, representing a compensatory mechanism to gather larger feedback information compared to PWTF (Watkins et al., 2016). If PWS do slow their speech as a compensatory mechanism, then more phonologically complex speech would require greater compensation and result in even slower speech.

Recording kinematic movements of the articulators is difficult given the anatomy of the system. Unlike for kinematic analysis of trunk or limb movements, most speech articulators are inaccessible for standard recording techniques. Nevertheless, the aforementioned evidence has been revealed using a wide variety of innovative methods. A commonly used method is electromagnetic articulography, in which small sensors are glued onto the lips, tongue, jaw, and (rarely) velum. Participants then sit in a magnetic field, and the positions of the sensors are tracked at very high temporal resolution (200 Hz). Other methods include infrared light-emitting diodes, which track movements from sensors placed on the skin (most commonly lips, as used in Smith et al., 2010); ultrasound, which places a probe beneath the jaw and is used to visualize tongue movements; and electromyography, which measures the excitability of the muscles involved in speech (i.e., the power of muscle contraction). Some of these methods are necessarily limited to measurement of one or two articulators at a time and require attaching recording equipment (such as electrodes) to articulators either within the vocal tract or externally, for example, on the lips. The necessary attachment of recording devices to the articulators alters sensations and potentially interferes with feedback processes during speech production. In PWS, altering somatosensory feedback can enhance fluency (Snyder et al., 2009), which could be problematic for interpretation of findings, and often restricts measurement to fluent speech. A noninvasive imaging technique, such as magnetic resonance imaging (MRI) of the vocal tract, has the advantage of allowing full examination of speech motor control in PWS without disturbing feedback or the actual movements themselves and, therefore, has the potential to capture movements involved in the production of disfluent speech.

Vocal tract MRI (vtMRI) offers the opportunity to view the movements of the entire vocal tract, from larynx to lips at good temporal (10–100 Hz) and spatial resolution (1–2 mm^2^; Carey et al., 2017; Kim et al., 2014; Niebergall et al., 2013; Ramanarayanan et al., 2013). A single image of the midline of the vocal tract (midsagittal slice) can be recorded in real time, producing two-dimensional video data that capture the fast movement of all the articulators simultaneously during speech. vtMRI is unique because it allows us to measure the range of movements of different articulators simultaneously, in one modality, providing novel information about the coordination of the articulators. Of particular interest is the ability to assess movement of articulators that are difficult to attach electrodes to, such as the velum. In contrast to techniques that attach sensors to specific flesh points, vtMRI can measure the contours along the entire length of the articulators, for example, along the tongue.

vtMRI has been used to inform linguistic theory and in clinical research. Within the field of linguistic theory, vtMRI has been used to study articulation in different languages (Carignan et al., 2015; Teixeira et al., 2012), coarticulation (Demolin et al., 2002), and consonant production in click languages (Proctor et al., 2014). vtMRI has also been used to investigate nonspeech events, such as beatboxing (Greer et al., 2018; Proctor et al., 2013) and swallowing (Zhang et al., 2012). vtMRI can be used to address important clinical questions. For example, vtMRI has been used to image patients who have undergone glossectomy (partial removal of the tongue) to treat oral cancer (Mády et al., 2003) and in a patient with apraxia of speech (Hagedorn et al., 2017). In the latter study, vtMRI was used to image gestural coordination throughout the vocal tract, as well as unphonated intrusion errors and gestures prior to the onset of speech. These studies used a range of MRI acquisition and analysis techniques. For example, acquisition speeds range from 10 to 100 Hz (with accompanying ranges in spatial resolution), and analysis techniques include region-of-interest analysis, which extracts information on the movement of individual articulators; grid-based analysis, which creates contours of the entire air–tissue boundary; or anatomy-guided techniques to segment individual articulators (see Ramanarayanan et al., 2018, for a review).

In the current study, we used vtMRI to scan the vocal tracts of a large sample of PWS and PWTF during speech production. Participants produced several repetitions of pseudowords increasing in syllable length from one to three syllables and four-syllable pseudowords that differed in complexity (Smith et al., 2010). We first measured lip aperture variability during fluent repetitions of pseudowords with an aim to reproduce the pattern of previous findings in PWS for this articulator that were measured using infrared light-emitting diodes attached to the lips (Smith et al., 2010). We next extended our analysis to measure movements of two additional articulators, the tongue body and velum. It is worth noting that the current approach was not a methodological replication as we adapted our methods in a number of ways for data collection inside the MRI scanner. First, as in the previous study, participants heard the pseudoword target (here spoken by the researcher) and repeated it. Each pseudoword was demonstrated and repeated until it was repeated accurately by the participant. During MRI scanning, pseudoword production was cued visually by the written form of the stimulus appearing on a screen, rather than presented auditorily. Second, pseudowords were produced singly rather than embedded in a carrier phrase, thereby limiting the amount of speech and head movements inside the scanner and shortening scanning times. Using a carrier phrase allowed segmentation of the movement *toward the* “m” sound in the previous study. However, it also increased variability of repeated productions (Kleinow & Smith, 2000). Without the carrier phrase, we chose to use the movement *out of* the “m” sound (i.e., the release of the lip closure) to identify the beginning of the pseudoword for analysis (see the Analysis Procedure section). Third, the sampling rate for data collection using MRI was 33.3 frames per second, whereas the method used in the previous study sampled movements at 250 Hz. Relatedly, we measured movement variability in space and time using the coefficient of variation (CoV) of the size of the movements in terms of time and amplitude, whereas the previous study normalized the movement trajectories for time and amplitude to produce the spatiotemporal index (STI) of variability. We discuss our results with consideration to these changes.

Our study aimed to demonstrate the feasibility of vtMRI to record speech motor control in both typical and diverse speaker populations. Specifically, we chose to measure the variability of speech movements during simple and complex utterances in PWS and PWTF, since this is the most reliable effect in previous kinematic studies (e.g., Smith et al., 2010). We also demonstrate one of the main benefits of vocal tract imaging, namely, the ability to capture information from multiple articulators, by measuring movement variability in the lips (as previously studied) and two additional articulators, the tongue body and velum, during the same utterances. Furthermore, we examined whether variability in speech movements was related to stuttering severity. Finally, given the lack of consensus on whether speech movement durations differ in PWS, we explored whether increasing syllable number or phonological complexity differentially affected movement durations in PWS and PWTF.

## Method

### Participants

We scanned 31 adults who stutter and 20 typically fluent controls. Data from one PWS were excluded due to technical reasons. Data from a further two PWS were excluded because fewer than six out of 10 utterances for each pseudoword were produced fluently during the scan (see the Analysis Plan section). This resulted in a sample of 28 adults who stutter (seven women and 21 men, *M*_age_ = 30.57 years, range: 19–45 years) and 20 controls (four women and 16 men, *M*_age_ = 29.4 years, range: 20–44 years). Groups were balanced for gender, age, ethnicity, and years of education (see Table 1). All PWS had at least mild stuttering severity, as assessed by the Stuttering Severity Instrument–Fourth Edition (SSI-4; see Table 1). Participants reported normal or corrected-to-normal vision and normal hearing. Exclusion criteria included any neurological impairment or disorder of speech, language, or communication other than developmental stuttering.

**Table 1.** Participant information.

Variable	PWS			PWTF		
	Range	*Mdn*	IQR	Range	*Mdn*	IQR
Age (years)	19–45	29.5	25–34	20–44	28.5	25.25–32.75
Education (years)	10–22	17	14–18	13–24	18	17–19.75
SSI-4 score	16–40	28.5	22.25–31			
Age of stuttering onset (years)	3–10	4	3–6			

*Note.* PWS = people who stutter; PWTF = people who are typically fluent; IQR = interquartile range; SSI-4 = Stuttering Severity Instrument–Fourth Edition.

Participants' speech was assessed using the SSI-4 (Riley, 2009). This instrument measures the frequency and duration of stuttered moments and physical concomitants. Participants were recorded in person with video. One person's speech was recorded via teleconference due to technical reasons. Participants read a passage and had a conversation with the researcher, each for 2 min. Recordings were scored off-line.

All PWS reported the onset of stuttering during childhood (i.e., before 10 years old). Twenty-six of the 28 PWS reported that they had visited a speech and language therapist at some point, but many were unable to recall specific details from their childhood experiences. The reported durations of therapy ranged from a few months to several years and involved learning a wide variety of techniques to help manage their stutter, most commonly breathing techniques and cognitive/acceptance therapies. Nine PWS reported that they continued to use the techniques at least occasionally. One PWS reported receiving therapy that specifically targeted speech rate but that this technique was no longer used. Participants were asked not to use these techniques during the task. No participants had received therapy within the last 6 months.

The University of Oxford Central University Research Ethics Committee (R52173/RE005) approved the study. Participants gave informed written consent to participate in the study, in accordance with the Declaration of Helsinki, and with the procedure approved by the committee.

### Experimental Procedure

Prior to scanning, a researcher demonstrated how the pseudowords were pronounced, and participants practiced them aloud until they were accurate. This was achieved usually after three repetitions of the pseudoword set.

The pseudoword stimuli were those used by Smith et al. (2010); three pseudowords of increasing length from one to three syllables (“mab” /mæb/, “mabshibe” /mæbʃaIb/, and “mabfieshabe” /mæbfaIʃeIb/) and two 4-syllable pseudowords with contrasting phonological complexity (“mabshaytiedoib” /mæbʃeitaIdɔIb/ and “mabteebeebee” /mæbtibibi/). Pseudowords started with a bilabial sound. This was important for the analysis of lip aperture and identification of the start of the utterance.

During scanning, each pseudoword was read 10 times, in a random order. For each trial, the pseudoword was displayed on a screen, and participants read it aloud at their natural speaking rate. Each trial lasted 3.5 s. In total, there were 50 trials resulting in a total scan run time of approximately 3 min.

### MRI Acquisition

Data were collected on a 3-T MRI system (Prisma, Siemens) using a 64-channel head and neck receive array. Midsagittal images of the vocal tract from lips to larynx were acquired with in-plane spatial resolution of 2 mm × 2 mm using a radial FLASH sequence (echo time/repetition time = 1.4/2.5 ms) with golden angle sampling. Images were reconstructed at 33.3 frames per second using a second-order spatiotemporal total generalized variation constraint (Knoll et al., 2011).

### Analysis Procedure

**Figure 1.**
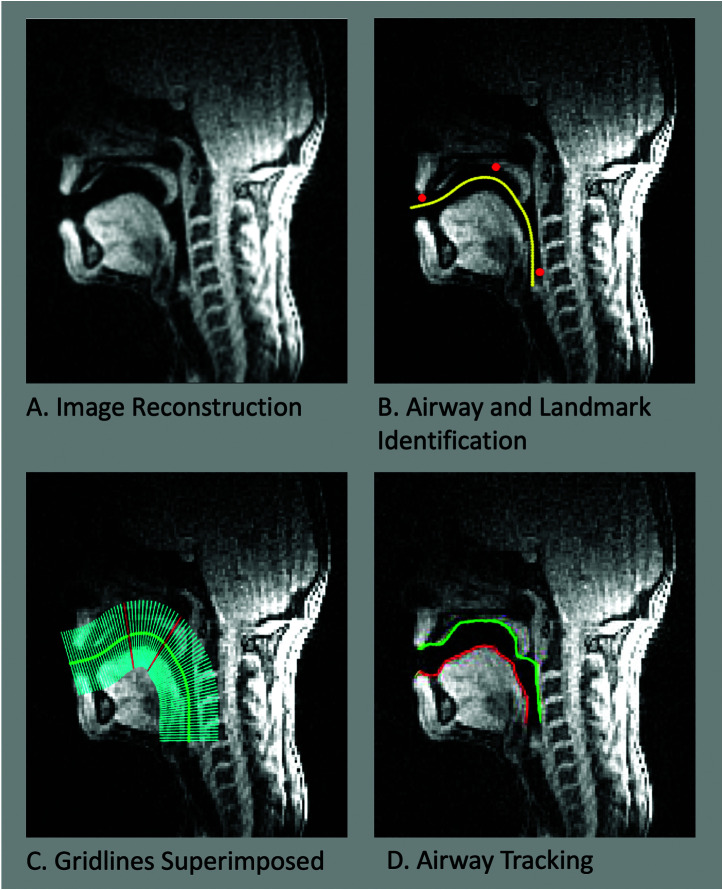
Image analysis pipeline. (A) Example (single frame) of the reconstructed image. (B) Using the air–tissue boundary toolbox (Kim et al., 2014), the airway was identified manually by drawing a line through the open vocal tract (yellow line). The lowest point of the upper lip, back of the hard palate, and larynx were also identified manually (red dots). (C) Equally spaced gridlines were placed orthogonal to the yellow line and centered on it. Gridlines highlighted in red were the ones used for tracking the tongue body and velum separately (see text). (D) Tracking of air–tissue boundaries. Upper boundary shown in green; lower boundary shown in red.

The imaging data were reconstructed into a video format and analyzed using a custom MATLAB toolbox that uses grid-based air–tissue boundary segmentation to track movements within the vocal tract (Kim et al., 2014). The schematic below shows the analysis pipeline (see Figure 1).

Using the air–tissue boundary toolbox (Kim et al., 2014), the airway was identified manually by drawing a line through the open vocal tract (see yellow line in Figure 1B). The lowest point of the upper lip, back of the hard palate, and the larynx were also identified manually (see red dots in Figure 1B). These points were used to guide the positioning of the grid. Gridlines were placed orthogonal to the midline of the vocal tract (see green line in Figure 1C) at 2-mm intervals. The intersections of the upper and lower air–tissue boundaries with each gridline were identified based on an abrupt change in pixel intensity (where white pixels, tissue, met black pixels, air), interpolated, and smoothed to create two continuous boundary lines (see red and green lines in Figure 1D). The segmentation performance of this toolbox was evaluated previously against manually annotated air–tissue boundaries for a range of phonemes. The root-mean-square error (of the Euclidian distance between manual and toolbox boundary points) was less than one pixel (0.71–0.93) across the entire vocal tract (Kim et al., 2014). The advantage of using automatic techniques, such as this one, to analyze large data sets, comprising thousands of frames per subject, clearly outweighs this degree of error.

### Lip Aperture Measurement

**Figure 2.**
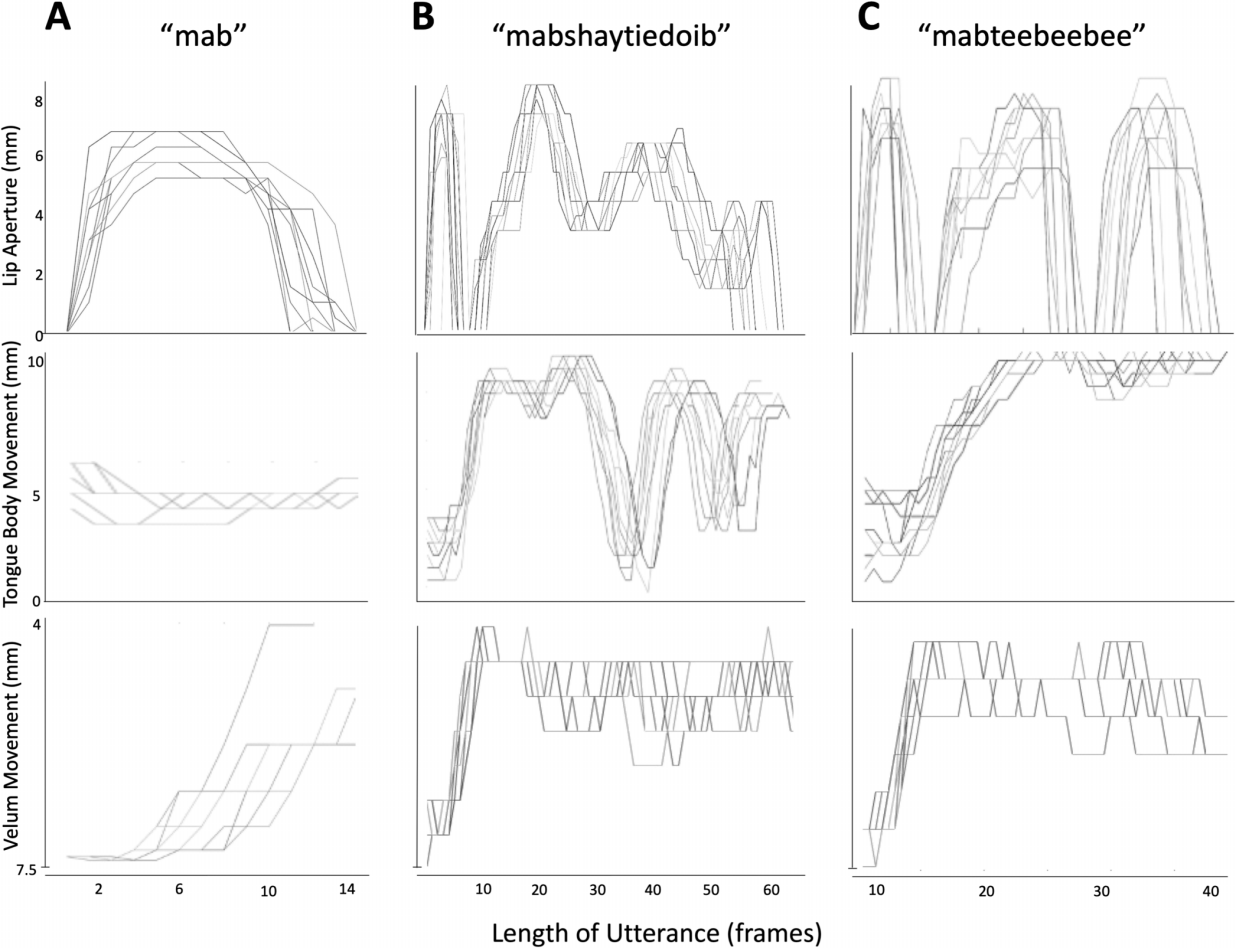
Examples of movement traces. Each plot shows 10 repetitions of the words (A) “mab,” (B) “mabshaytiedoib,” and (C) “mabteebeebee” for a single representative participant. Each line represents one repetition. The start and end points are defined as the frame where lip aperture departs from zero for the /m/ and returns to zero for the final /b/, respectively.

The distance between the first points along the upper and lower air–tissue boundaries gave the lip aperture in millimeters (see Figure 1). The start of the utterance was identified as the latest time frame at which the lip aperture was zero for the /m/ sound (i.e., the release of the bilabial). The end of the utterance was identified as the time frame that the lip aperture first returned to zero for the final bilabial closure of the word, for example, /b/. An example of the lip aperture traces is shown in Figure 2.

Variability was calculated using the CoV, that is, the standard deviation of the size of the movements across 10 repetitions of each word, divided by the mean. The size was simply the sum of the aperture of the movements across frames capturing both the amplitude and duration of the movement. Movement duration was also averaged for each repetition by summing the total number of frames from the start to the end of the utterance as defined above.

### Velum and Tongue Body Measurements

Velum and tongue movements were measured in a similar way. For the tongue body, the position of the lower air tissue boundary (shown in red in Figure 1D) was tracked as it moved along a single gridline. We selected the gridline that was closest to the highest point of the dorsal boundary of the tongue body in the frame where the tongue reaches its most dorsal extent during the first /i/ sound of “mabteebeebee.” The lowest position of the tongue body along this gridline from the entire scan was selected as a reference point to which all frames were compared. For each frame, we measured the Euclidian distance from this reference point along the gridline to the position of the tongue body.

For the velum, the upper air–tissue boundary (ventral surface of the velum; shown in green in Figure 1D) was tracked up and down a single gridline. This gridline was chosen as the closest to the middle of the velum, where the velum is seen to bend when raised, which corresponds to the position with the largest range of velum movement. The point at which this part of the velum was highest in the entire scan was selected as a reference point for the measurements made along this gridline in all other frames.

For the tongue body and the velum, the start and end frames of each utterance were the same as those used for the lip described above. Examples of the tongue body and velum movements are shown in Figure 2. The CoV for tongue and velum movements was determined as for the lip aperture.

### Analysis Plan

Trials resulting in exclusion were rare. If a participant did not produce at least six (out of 10) fluent and accurate productions of a pseudoword, data for that pseudoword were excluded from analyses. Trials containing stuttering will be used in future work. Two full data sets (PWS) were excluded prior to analysis based on this criterion. Six PWS and two PWTF had partial data sets (excluded data for one or more of the words). In total, 5.7% of words from the stuttering group and 4% of words from the control group were excluded. The exclusions were considered to be missing at random. Excluded data are visualized in Figure 4.

We used linear mixed models (lme4 package in R; Bates et al., 2015) to model interactions between group, word, and articulator with subject included as a random factor. Importantly, linear mixed models are robust to a small amount of random missing data, allowing us to use data from nearly all our participants (Krueger & Tian, 2004).

Two linear mixed models were used to capture between-groups differences in variability of speech movements in three separate articulators in relation to (a) word length (one to three syllables) and (b) phonological complexity (four-syllable complex and simple). Models included participant as random factor. For the group comparisons relating to duration, we used two additional models that did not include articulator as a fixed factor.

Main effects and interactions are reported using the *anova* command from the base R stats package (R Core Team, 2019) with Type III analysis of variance using Satterthwaite's method (Luke, 2017). Where appropriate, *t* tests were used to assess specific contrasts. For these analyses, partial eta squared and Cohen's *d* effect sizes were calculated using the *effectsize* package (Ben-Shachar et al., 2020). Full models (with comparisons between each factor for categorical variables) are shown in Supplemental Materials S1–S4. Marginal and conditional *R* ^2^ were calculated to represent the variability accounted for by the fixed effects alone and the fixed and random effects in the model, respectively. Normalized beta estimates (β) were calculated using the *tab_model* function of the *sjplot* package in R (Lüdecke, 2019) to facilitate comparison of effect sizes across the independent variables within each model.

### Data Availability

The derived data associated with this article are available on the Open Science Framework at https://osf.io/3qdnv/. The MRI acquisition sequence and reconstruction code are available upon reasonable request to author Mark Chiew (mark.chiew@ndcn.ox.ac.uk).

## Results

### Movement Variability

**Figure 3.**
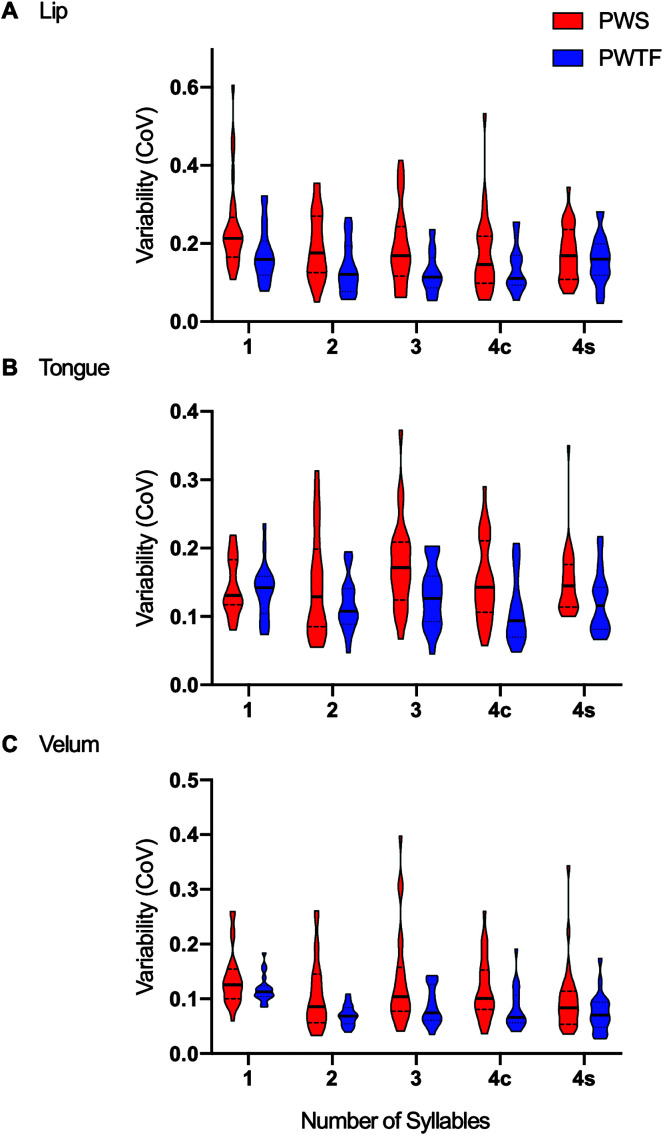
Variability of articulator movements over repeated utterances of the pseudoword set. CoV = coefficient of variation; PWS = people who stutter; PWTF = people who are typically fluent; 4c = four-syllable, complex word (“mabshaytiedoib”); 4 s = four-syllable, simple word (“mabteebeebee”). Violin plots are shown to visualize the distribution of data and its probability density for each group separately for each syllable set. Solid horizontal lines represent the median, and dashed lines show the interquartile range.

**Figure 4.**
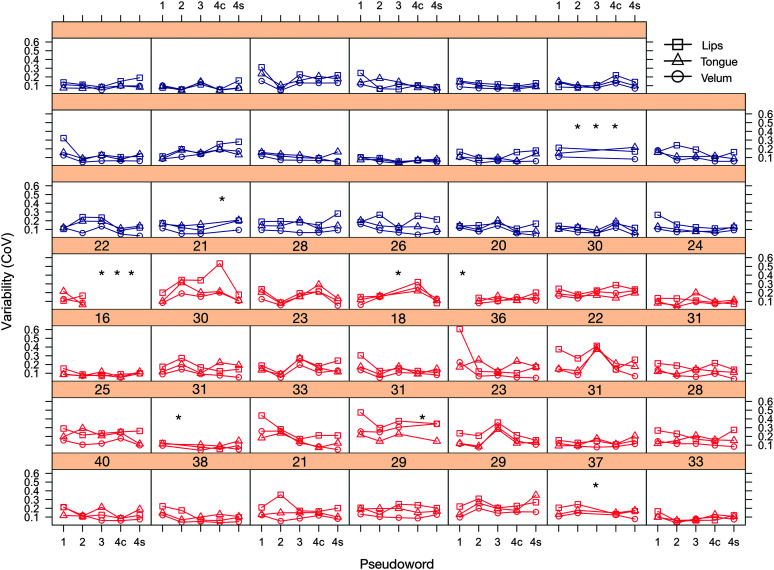
Individual variability scores for pseudowords with one to three syllables and the complex (4c) and simple (4s) four-syllable pseudowords. Red participants = people who stutter; blue participants = people who are typically fluent. Data from some participants are missing due to speech errors (see the Analysis Plan section). SSI scores are shown above individual data plots for people who stutter. * indicates data missing for one pseudoword. CoV = coefficient of variation.

The amount of variability (CoV) for each pseudoword and participant in the two groups is plotted for each articulator in Figure 3. The pattern of results across all articulators for each individual participant is shown in Figure 4.

We examined whether variability in speech movements during fluent repetitions of pseudowords differed between PWS and PWTF using two separate linear mixed-effects models. The dependent measure was the coefficient of variability for movement sizes in three different articulators for repetitions of (a) pseudowords of different syllable lengths (one, two, and three syllables) or (b) four-syllable pseudowords of different phonological complexity (simple and complex). Fixed-effect terms in each model included group (PWS vs. PWTF), word (either one, two, and three syllables, or simple vs. complex), articulator (lips, tongue, and velum), and the Group × Pseudoword, Group × Articulator, Pseudoword × Articulator, and Group × Pseudoword × Articulator interactions. The random-effects terms included participant and the interaction of participant with the fixed-effects terms of group, word, and articulator.

#### The Effect of Pseudoword Length on Variability

The overall model predicting variability had a total explanatory power (conditional *R* ^2^) of 69.79%, in which the fixed effects explained 26.05% of the variance (marginal *R* ^2^). Within this model, the main effects of group, word, and articulator were significant. In addition, there was a significant interaction between group and articulator as well as between word and articulator. These interactions were explored using the full model results, which are presented in Supplemental Material S1.

PWS had significantly greater variability in their speech movements than PWTF (significant main effect of group, *F*(1, 45.95) = 10.47, *p* = .002, η_p_^2^ = .19). This group difference was greatest for lip compared with tongue (*p* < .001*,* β = .44) and velum (*p* = .005, β = .33) movements, which showed a similar size group difference (significant interaction between group and articulator, *F*(2, 262) = 6.05, *p* = .003, η_p_^2^ = .04). Follow-up analyses showed that PWS had greater variability than PWTF for all articulators: lip, *t*(132.9) = 4.32, *p* < .0001, *d* = 0.70; tongue, *t*(133.4) = 2.98, *p* < .01, *d* = 0.48; velum, *t*(119.7) = 3.73, *p* < .001, *d* = 0.59. For both groups, speech movement variability was greatest for repetitions of the one-syllable pseudoword relative to the two-syllable (*p* < .001, β = .10) and three-syllable (*p* < .002, β = .22) pseudowords, which did not differ (main effect of word *F*(2, 44.04) = 5.33, *p* = .008, η_p_^2^ = .19). Movement variability was greatest for the lip relative to the tongue (*p* < .001, β = .33) and velum (*p* < .001, β = .09) movements, which did not differ (main effect of articulator, *F*(2, 262) = 81.40, *p* < .001, η_p_^2^ = .38). These last two factors interacted significantly (Word × Articulator interaction, *F*(4, 262) = 6.60, *p* < .001, η_p_^2^ = .09), due to a more pronounced syllable length effect in the lip movements relative to movements of the tongue (*p* < .001, β = .29) and velum (*p* = .003, β = .36).

#### The Effect of Phonological Complexity on Variability

The overall model predicting variability had a total explanatory power (conditional *R* ^2^) of 58.72%, in which the fixed effects explained 20.98% of the variance (marginal *R* ^2^). Within this model, the main effect of group was significant. In addition, there was a main effect of articulator, but there were no significant interactions. The full model output is presented in Supplemental Material S2.

PWS had significantly greater variability in their speech movements than PWTF (significant main effect of group, *F*(1, 39.5) = 6.08, *p* = .018, η_p_^2^ = .13), and this group difference was seen for the movements measured in lip, tongue, and velum (interaction with articulator was not significant). For both groups, speech movement variability was greatest for the velum relative to the tongue (*p* = .008, β = .11) and lip (*p* < .001, β = .11) movements, which did not differ (main effect of articulator, *F*(2, 174) = 48.71, *p* < .001, η_p_^2^ = .19). Phonological complexity did not affect speech movement variability in either PWS or PWTF or in any of the articulators measured (main effect of word was not significant and did not interact with any other factor).

#### The Relationship Between Movement Variability and Stuttering Severity

In addition, the relationship between lip movement variability data and SSI was assessed. We selected the pseudoword “mabshaytiedoib” a priori for this analysis, as it is the most complex and the one that we predicted would show the greater variability. There was no correlation between variability score and SSI score (*r* = −1.39, *p* = .177). As our hypothesis that more complex pseudowords would be repeated with greater variability compared with simple pseudowords was not upheld, we further explored the relationship between SSI score and overall variability (average across Pseudowords 1–3). Again, there was no correlation between SSI score and variability (*r* = .01, *p* = .95).

#### Summary of Variability Analyses

In summary, PWS had greater variability relative to PWTF in lip, tongue, and velar movements during fluent productions of pseudowords. Increasing pseudoword length and phonological complexity did not differentially affect speech movement variability in PWS compared with PWTF. All effects were most pronounced for lip aperture movements relative to the variability measurements of movements of the other articulators measured: the tongue body and the velum. Variability was maximal for repetitions of one-syllable pseudowords. There was no relationship between variability during fluent productions of pseudowords and stuttering severity.

### Movement Duration

**Figure 5.**
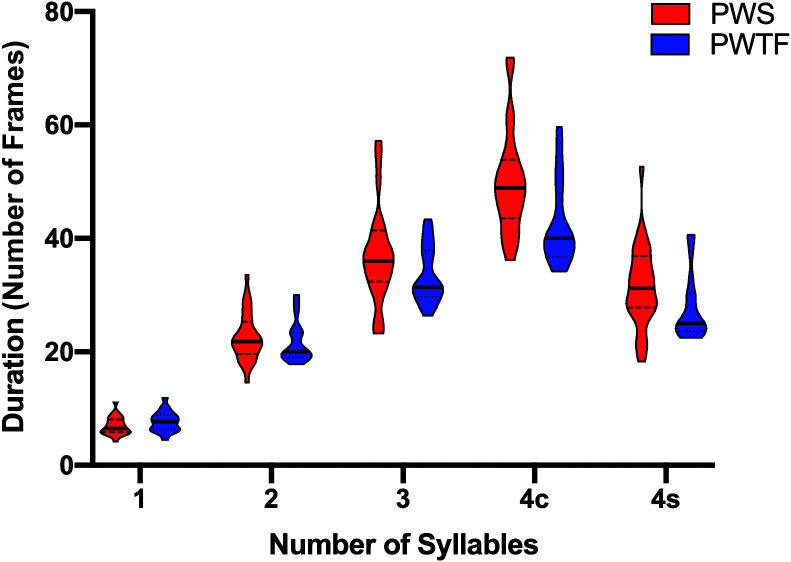
Duration of responses. PWS = people who stutter; PWTF = people who are typically fluent; 4c = four-syllable, complex word (“mabshaytiedoib”); 4s = four-syllable, simple word (“mabteebeebee”). Violin plots are shown to visualize the distribution of data and its probability density for each group separately for each syllable set. Solid horizontal lines represent the median, and dashed lines show the interquartile range.

The duration of responses for repetitions of each pseudoword and participant in the two groups is plotted in Figure 5. We examined whether movement durations during fluent repetitions of pseudowords differed between PWS and PWTF using a linear mixed-effects model to compare pseudowords of different syllable lengths. A similar model was run for the four-syllable words to compare phonological complexity. Fixed-effect terms in each model included group (PWS vs. PWTF) and word (either one, two, and three syllables, or simple vs. complex), and the Group × Word interactions. The random-effects terms included participant and the interaction of participant with the fixed-effects terms of group and word.

#### The Effect of Pseudoword Length on Movement Duration

The overall model predicting duration had a total explanatory power (conditional *R* ^2^) of 93.79%, in which the fixed effects explain 85.67% of the variance (marginal *R* ^2^). Within this model, the main effect of word was significant (note that this was highly expected as words with more syllables were expected to have longer durations). In addition, there was a significant interaction between group and word (see Figure 5). This interaction was explored using the full model results, which are displayed in Supplemental Material S3. The main effect of group was not significant.

PWS had significantly longer speech movement durations than PWTF when repeating the two-syllable (*p* = .017, β = .05) and three-syllable (*p* < .001, β = .12) words compared with the one-syllable word, and the three-syllable pseudoword compared with the two-syllable pseudoword (*p* < .001, β = .07; significant interaction between group and word, *F*(2, 361.8) = 15.6, *p* < .001, η_p_^2^ = .08; see Figure 5). As expected, for both groups, movement durations were longest for repetitions of the three-syllable pseudoword compared with the two-syllable (*p* < .001, β = .5) and one-syllable (*p* < .001, β = 1.12) pseudowords and were longer for the two-syllable pseudoword compared with the one-syllable pseudoword (*p* < .001, β = .63; significant main effect of word, *F*(2, 361.8) = 2618.1, *p* < .001, η_p_^2^ = .94).

#### The Effect of Phonological Complexity on Duration

The overall model predicting duration had a total explanatory power (conditional *R* ^2^) of 94.37%, in which the fixed effects explained 56.79% of the variance (marginal *R* ^2^). The full model results are displayed in Supplemental Material S4. PWS had significantly longer movement durations than PWTF for repetitions of four-syllable pseudowords (*p* = 0.004, β = .26; main effect of group). This group difference was significantly more pronounced for repetitions of the complex relative to the simple pseudowords (*p* < .001, β = .09; significant interaction between group and word). For both PWS and PWTF, speech movement durations were significantly longer for repetitions of the complex relative to the simple four-syllable pseudowords (*p* < .001, β = .77; main effect of word).

#### Summary of Duration Analyses

In summary, PWS show longer speech movement durations relative to PWTF. These group differences emerge only for repetitions of multisyllabic pseudowords and were even more pronounced when the phonological complexity was increased. Expectedly, durations were longer for both groups when the number of syllables or the phonological complexity increased.

## Discussion

### Summary of Findings

We tested whether there were differences in articulator movements during perceptually fluent speech between PWS and PWTF. We used a novel method, MRI of the vocal tract, to capture movement of the lips, tongue body, and velum in 28 PWS and 20 PWTF as they repeated pseudowords. The pseudowords were designed to determine the effects of word length (one to three syllables) and phonological complexity (Smith et al., 2010). We found differences in the variability of articulator movement and duration of responses between PWS and PWTF. Overall, the stuttering group repeated the utterances with more variability than the control group, but this effect did not interact with pseudoword length or phonological complexity. These findings are in accord with those from previous investigations, showing a greater amount of variability in the fluent speech movements of PWS compared with PWTF (Frisch et al., 2016; Howell et al., 2009; Jackson et al., 2016; Loucks & De Nil, 2006, 2012; Loucks et al., 2007; Sasisekaran, 2013; Smith et al., 2010). We found no relationship between movement variability and stuttering severity, however.

In contrast with previous findings, our analysis revealed higher variability scores for the one-syllable relative to the two- and three-syllable pseudowords. It is possible that this difference is explained by our lower sampling rate or our measure of variability compared with those used in previous studies and reflects a limitation of our study. On the other hand, the extension of our analysis to explore movements of articulators other than the lips during production of the same words demonstrates one of the advantages of vtMRI. Our analysis revealed that variability was also greater in PWS compared with PWTF for movements of the tongue and velum, though these effects were smaller than those seen for the lips, for which the specific stimulus set used here was designed.

Previous findings on whether duration of movements differs in PWS were mixed (McClean & Tasko, 2004; Smith et al., 2010, 2012; Tasko et al., 2007; Usler et al., 2017). Here, our analysis also revealed a proportionally greater effect of word length and phonological complexity on duration in PWS compared with PWTF. Both groups took longer to repeat pseudowords as syllable number and phonological complexity increased, but this effect was significantly more pronounced in PWS compared with PWTF.

Below, we discuss each of these findings in turn before outlining some of the limitations of our study that relate, in part, to differences between the different methods used to measure vocal tract movement. Finally, we briefly discuss some future directions and implications for the use of vtMRI in the study of movement control in PWS.

### Greater Speech Movement Variability in PWS

The main finding of the current study was confirmation of greater movement variability in PWS during fluent speech as previously reported using a variety of other methods (see introduction and references therein). This pattern of instability in speech motor control is thought to reflect noise, manifest as signal delays, in the cueing of articulatory sequences used to execute the planned sequence of assembled phonemes. Variability of speech movements could be interpreted in a number of theoretically important ways. One possibility is that stuttering is caused by a discrepancy between the expected utterance and the actual utterance produced (Bohland et al., 2010; Guenther, 2016; Max et al., 2004) caused by an error in the predicted movement, the actual movement, or less tolerance of a typical range of error. Our data support the hypothesis that the actual movements are more variable, which leads to greater chance that the sensory–motor feedback will not match with a predicted response resulting in an error signal. The error signal generated may cause an inhibitory response, leading to a block, repetition, or prolongation of the sound. Thus, even though our analysis was restricted to fluent utterances, it is hypothesized that more variable movements increase the likelihood that the system will act to inhibit speech.

### Variability and Stuttering Severity

Importantly, many PWS in the current study had levels of variability that were within the range of PWTF. This heterogeneity was also described previously (Smith et al., 2010), and means that increased variability cannot be considered a diagnostic characteristic of developmental stuttering. Instead, there could be subtypes within PWS whereby reduced control over the articulators is characteristic of a subset of PWS only. Alternatively, this instability in the speech motor control system during fluent speech production could manifest inconsistently. These potential subgroups were not explained by severity of stuttering, as there was no relationship between severity (SSI score) and variability.

The lack of a linear relationship between the amount of variability and stuttering severity in our data could be explained by the fact that SSI measures a range of characteristics of stuttering, including duration of stuttered moments, and characteristics of physical concomitants. In addition, stuttering severity is known to be affected by factors beyond speech motor control, such as learned anxiety in response to stuttering (Alm, 2014). It is important to remember that the increased variability was observed during fluent speech production, so perhaps a relationship with stuttering symptoms should not be expected. In addition, it is unknown how stable these patterns of motor instability are within individuals. Repeated assessments of the same individuals would shed light on the reliability of this motor characteristic.

### The Effect of Complexity on Duration of Movements

The relationship between variability and severity may be further complicated by compensatory strategies. For example, PWS may reduce their speech rate in order to maintain fluency (Andrews et al., 1982). As greater demands are placed on the speech motor system, it could be that PWS compensate by slowing down their speech (Max et al., 2003; Peters et al., 1989; Van Lieshout et al., 1996). Our data support this hypothesis: Some PWS produced utterances with longer durations than PWTF, but only when the pseudowords became more complex (either due to more syllables or phonological complexity). Slowing speech rate would allow accumulation of evidence from feedback (sensory reafference; Watkins et al., 2016). This may be an automatic response at the neural level or could represent a conscious effort to maintain fluency. Fluency-enhancing techniques such as altering auditory feedback, choral speaking, and singing all typically involve slower production, and speech and language therapies often focus on slowing speech rate in order to improve fluency. It is possible that some PWS consciously slow down their speech when the utterance becomes more difficult, but it is more likely that this is an implicitly acquired consequence of producing more complex speech. Future studies should examine the effect of slowing down speech rate on variability in PWS.

### The Lack of an Effect of Complexity on Variability

The results of the current study did not reveal the expected effect of complexity (syllable number or phonological complexity) on the variability of speech movements across repeated utterances. Previous studies found that the inconsistency in coordinated speech movements increased with phonological complexity to a greater extent in PWS than in PWTF (Kleinow & Smith, 2000; Smith et al., 2010; Soderberg, 1966). Our findings suggest, therefore, that the complexity of the utterance does not impact the degree of control over the speech motor system and does not modulate the degree of “noise” in the speech motor system, which is thought to contribute to increased likelihood of disfluency. However, before reaching this conclusion on the basis of negative evidence (failure to find an effect), it is worth considering a related surprising effect revealed by our analysis. We found a main effect of pseudoword length that was driven by higher variability for production of the shortest compared with longer pseudowords. This effect was seen in both groups and did not differ between them. This was contrary to previous findings and our expectations, which were that the shortest pseudoword would have the least amount of variability compared with longer pseudowords. The differences in measurement may explain these discrepancies and are discussed in further detail below. It would be important to replicate this finding before concluding that complexity and speech movement variability are unrelated in PWS.

### Variability Throughout the Vocal Tract

In addition to measuring the lip aperture using vtMRI to replicate previous work, we aimed to measure the movement of articulators that were previously difficult to measure noninvasively due to their positioning within the vocal tract (tongue and velum). This exploits the benefits of vtMRI. In addition to the lip aperture, we also measured variability for the tongue body and velum. We found that movement variability was greater in PWS compared with PWTF for the tongue and velum for production of the four-syllable pseudowords; in the analysis of the one- to three-syllable pseudowords, the group difference was maximal for the lips (significant interaction) but was also significant for the tongue and velum. These results are consistent with those from a previous study, which found greater variability in PWS compared with PWTF using ultrasound to measure stability of movements of the tongue and velum over repetitions of simple utterances (Frisch et al., 2016). There was a strong correlation between the amount of variability for each of the articulators, but overall, there was less variability for velar movements compared with both lip and tongue movements. This effect of articulator is most likely due to the different involvement of the articulators in each of the utterances of the stimuli used in this study. The pseudowords were taken from a previous study and contained primarily bilabial sounds, as the focus of measurement in that study was on lip movements. While using a well-studied set of stimuli allows us to compare the pattern of results between studies, the limited number of nasals in this specific pseudoword set reduced the amount of velum movement required to produce the utterances (e.g., the velum is only critically involved in the sound /m/; see Figure 2). A more focused study of velar movements would be informative. Future studies should choose stimuli with a larger range of phonemes. Overall, our results suggest that variability generalizes across articulators (e.g., if participants had high variability for the lips, they were likely to have high variability for the velum and tongue, as well).

### Limitations

The aim of the current study was to determine whether vtMRI could reproduce the findings of a previous study using infrared light-emitting diodes to measure lip aperture variability in PWS (Smith et al., 2010), specifically that movement variability was greater and disproportionately increased with stimulus complexity in PWS compared with PWTF. We were successful in part of this aim, as described above. Nevertheless, the implementation of this paradigm in the MRI scanner necessitated some important changes from the previous approach that may explain some discrepancies between the findings of the two studies. We summarized these changes in the introduction. Here, we discuss them further in the context our findings.

Stimuli were cued for production during scanning with a visually presented word. An auditory presentation would have provided a more precise target for production and encouraged imitation of the stimulus duration, in particular. Without such a template, greater variability in the duration of pseudowords could have occurred, and such variability would also increase with word length. Our measurement and analysis did not reveal increased variability with increasing word length, however. Furthermore, we cannot explain why such an effect would occur in PWS and not in PWTF without arguing that PWS produce more variable speech movements, which is concluded here. Even so, future studies should consider using an auditory presentation to reduce this potential source of variability.

As explained in the introduction, we did not use a carrier phrase during production of the target pseudowords inside the scanner. This resulted in a measurement difference in that we started our measurement of lip aperture from the last frame where the lips were closed, that is, the release of the bilabial constriction, rather than from the beginning of the lip closure as in the previous study. This difference would result in shorter stimulus durations and, coupled with a loss in resolution in the temporal domain due to the differences in sampling rate (33.3 frames per second vs. 250 Hz), less precise measurements. The precision of measurement of duration of particularly short bilabial closures, which can last between 60 and 200 ms (Löfqvist, 2006), would be considerably reduced at the lower rate (two to six frames in the current study cf. 15–50 samples in Smith et al., 2010). This imprecision regarding the start and end of the closures could unduly affect measurement of the shorter words with a higher proportion of bilabial sounds (e.g., “mab”) and potentially is the cause of the unexpected higher variability measurements seen across participants in production of the one-syllable relative to the multisyllable pseudowords.

A key difference in this study compared with the previous one (Smith et al., 2010) was the measure used to capture variability of movement. The previous study used the STI of variability (Smith et al., 1995), and here, we used the CoV. The STI was used in several previous studies involving analyses of kinematic and acoustic data with higher sampling rates (e.g., Howell et al., 2009; Jackson et al., 2016; Smith et al., 1995, 2010). The STI calculation used previously involved normalization of amplitude (*z*-score transformation) and time (resampling to 1,000 data points) in the movement trajectories in order to determine variability of the relative timing of articulator movements. The standard deviation was used to measure variation at a sample of 50 time points across the trace and summed to produce an index. Applying the STI calculation here would involve upsampling of our low (temporal) resolution vtMRI data. Therefore, we opted for the simpler calculation of the CoV, which captures variability in movement size, that is, amplitude by duration, and normalizes for the increased length of the word (as the standard deviation is divided by the mean of the utterance). In our view, the STI and CoV measures should be well correlated, but differences in their calculations might explain some of the differences in the findings of the two studies. The normalization procedures used in both calculations should result in comparable estimates of variability across words irrespective of length, but it is possible that upsampling for the single-syllable pseudowords and interpolation reduced variability estimates in the STI calculation. On the other hand, as described above, our low sampling rate applied to these short utterances might have overestimated variability using the CoV. Further work is needed to clarify the differences in these methods, especially when applied to short-duration stimuli.

A further limitation of this work is the unknown contribution of stuttering therapy on our measures of variability. As is typical of this population, nearly all of the participants reported that they had visited a speech and language therapist at some point in their lives. The reported type and duration of this therapy was very variable. Over half of participants reported prior experience with fluency enhancing techniques, and several reported that they continued to use these techniques occasionally. All participants were asked not to use techniques during the experiment.

### Future Directions and Clinical Implications

This is the first study to use vtMRI to measure speech movement variability and duration in PWS. While we demonstrate that the imaging and analysis techniques involved are sensitive enough to detect the finding of greater variability in PWS and PWTF, vtMRI offers many more opportunities to measure different aspects of speech motor control, with both clinical and theoretical importance. For example, variability, as measured here, gives a measure of the overall control of the speech motor system; however, more fine-grained analysis examining coarticulation and coordination and timing between articulators is also possible using vtMRI. This is particularly valuable when recording atypical speech or speech errors (e.g., apraxia of speech; Hagedorn et al., 2017). While the noise of the scanner may still enhance fluency in PWS, we have also recorded stuttered moments using vtMRI during cued (Lu et al., 2021) and spontaneous speech. Analyses of these data sets will help us to understand the precise timing of interarticulatory movement during stuttered speech, which is of relevance to models of articulation sequencing (e.g., Bohland et al., 2010; Kearney & Guenther, 2019; Tilsen, 2018).

Our analysis involved extracting movement information from small areas of the articulators (along a single gridline for each). Techniques such as functional principal components analysis (Hoole & Pouplier, 2017) or generalized additive mixed models (Carignan et al., 2020) can analyze the entire contour of the vocal tract over time. Application of such techniques may shed further light on differences among PWS or in comparison with other groups regarding clinical markers of fluent and stuttered speech and how these relate to a richer assessment of stuttering severity, as was found with fMRI data (Ingham et al., 2012). Other important aspects of stuttering, such as covert strategies, lived experience, and psychosocial factors could also be considered alongside a more comprehensive analysis of individual differences in the history of therapy.

A measure of variability during fluent speech, as used here, could be a useful tool for objectively assessing early indications of improvement from therapy. As shown here, vtMRI can measure differences in articulation without relying on overt moments of stuttering: Measuring articulation during childhood may also inform more accurate models to describe the factors involved in the emergence and continuation of stuttering. However, we think it is important to note that the accessibility of MRI to many clinicians may limit its use in routine care, and it is likely to remain primarily a research tool.

### Summary and Conclusions

In summary, we used vtMRI to show that PWS have greater variability in the movements of the articulators during fluent utterances compared with PWTF in accord with findings from previous studies (Kleinow & Smith, 2000; Smith et al., 2010). Furthermore, we extended our previous knowledge by exploiting the benefits of vtMRI to measure multiple articulators within the vocal tract. Our results show that vtMRI is sensitive to subtle differences in articulator movement between PWS and PWTF, even during perceptually fluent speech. This is the first study to use vtMRI to study speech movements in PWS, and we demonstrate the important contributions that this novel technique can make to future stuttering research.

## Supplementary Material

10.1044/2021_JSLHR-20-00507SMS1 Supplemental Material S1 Effect of word length on variability.

10.1044/2021_JSLHR-20-00507SMS2 Supplemental Material S2 Effect of phonological complexity on variability.

10.1044/2021_JSLHR-20-00507SMS3 Supplemental Material S3 Effect of word length on duration.

10.1044/2021_JSLHR-20-00507SMS4 Supplemental Material S4 Effect of phonological complexity on duration.
