# Supplementary material for: Speech Movement Variability in People Who Stutter: A Vocal Tract Magnetic Resonance Imaging Study
Source: J Speech Lang Hear Res. 2021 Jun 22;64(7):2438–52. doi: 10.1044/2021_JSLHR-20-00507 (PMC8323486; doi:10.1044/2021_JSLHR-20-00507)
Supplement: Supplemental Material S1 [file JSLHR-64-2438-s001.pdf]

Supplemental Material S1. Effect of word length on variability.

| | Variability (CoV) | | | |
|---|---|---|---|---|
| *Predictors* | *std. Beta* | *Estimates* | *CI* | *p* |
| (Intercept) | | 0.24 | 0.22 – 0.26 | <0.001 |
| Group PWS:PWTF | -0.14 | -0.07 | -0.11 – -0.03 | <0.001 |
| Word 1:2 | -0.10 | -0.05 | -0.08 – -0.02 | 0.001 |
| Word 1:3 | 0.22 | -0.05 | -0.09 – -0.02 | 0.002 |
| Word 2:3 | 0.05 | 0.03 | -0.01 – 0.06 | 0.102 |
| Articulator Lip:Velum | 0.09 | -0.10 | -0.13 – -0.08 | <0.001 |
| Articulator Lip:Tongue | -0.33 | -0.10 | -0.12 – -0.08 | <0.001 |
| Articulator Velum:Tongue | -0.09 | 0.01 | -0.02 – 0.03 | 0.611 |
| Group PWS:PWTF * word 1:2 | 0.08 | 0.02 | -0.03 – 0.06 | 0.490 |
| Group PWS:PWTF * word 1:3 | -0.64 | 0.01 | -0.04 – 0.06 | 0.778 |
| Group PWS:PWTF * word 2:3 | 0.23 | -0.01 | -0.06 – 0.04 | 0.688 |
| Word1:2 * Lip:Velum | 0.03 | 0.02 | -0.01 – 0.05 | 0.181 |
| Word1:3 * Lip:Velum | 0.36 | 0.05 | 0.02 – 0.08 | 0.003 |
| Word2:3 * Lip:Velum | -0.08 | 0.03 | -0.01 – 0.06 | 0.098 |
| Word1:2 * Lip:Tongue | -0.09 | 0.05 | 0.02 – 0.09 | 0.001 |
| Word1:3 * Lip:Tongue | 0.29 | 0.09 | 0.06 – 0.12 | <0.001 |
| Word2:3 * Lip:Tongue | 0.11 | 0.03 | 0.00 – 0.07 | 0.040 |
| Word1:2 * Velum:Tongue | 0.06 | 0.03 | -0.00 – 0.06 | 0.051 |
| Word1:3 * Velum:Tongue | 0.13 | 0.04 | 0.01 – 0.07 | 0.021 |
| Word2:3 * Velum:Tongue | 0.08 | 0.01 | -0.03 – 0.04 | 0.691 |
| Group PWS:PWTF * Lip:Velum | -0.33 | 0.05 | 0.02 – 0.09 | 0.005 |
| Group PWS:PWTF * Lip:Tongue | -0.44 | 0.06 | 0.03 – 0.10 | <0.001 |
| Group PWS:PWTF * Velum:Tongue | -0.12 | 0.01 | -0.02 – 0.05 | 0.489 |

| | | | | |
|---|---|---|---|---|
| Group PWS:PWTF * Word 1:2 * Lip:Velum | -0.61 | -0.03 | -0.08 – 0.02 | 0.189 |
| Group PWS:PWTF * Word 1:3 * Lip:Velum | 0.36 | -0.03 | -0.08 – 0.02 | 0.179 |
| Group PWS:PWTF * Word 2:3 * Lip:Velum | 0.00 | 0.00 | -0.05 – 0.05 | 0.968 |
| Group PWS:PWTF * Word 1:2 * Lip:Tongue | -0.09 | -0.04 | -0.09 – 0.01 | 0.168 |
| Group PWS:PWTF * word 1:3 * Lip:Tongue | 0.29 | -0.05 | -0.10 – -0.00 | **0.045** |
| Group PWS:PWTF * Word 2:3 * Lip:Tongue | 0.10 | -0.02 | -0.07 – 0.03 | 0.527 |
| Group PWS:PWTF * Word 1:2 * Velum:Tongue | 0.64 | -0.00 | -0.05 – 0.05 | 0.949 |
| Group PWS:PWTF * Word 1:3 * Velum:Tongue | 0.09 | -0.02 | -0.07 – 0.03 | 0.509 |
| Group PWS:PWTF * Word 2:3 * Velum:Tongue | -0.04 | -0.02 | -0.07 – 0.04 | 0.553 |

Random Effects

| | |
|---|---|
| Marginal $R^2$ | 0.261 |
| Conditional $R^2$ | 0.698 |
| $N_{participant}$ | 48 |
| Observations | 411 |

R formula = variability ~ group * word * articulator + (1 + word | p_code), REML = TRUE, contrasts = contra.sum)
