# Supplementary material for: Speech Movement Variability in People Who Stutter: A Vocal Tract Magnetic Resonance Imaging Study
Source: J Speech Lang Hear Res. 2021 Jun 22;64(7):2438–52. doi: 10.1044/2021_JSLHR-20-00507 (PMC8323486; doi:10.1044/2021_JSLHR-20-00507)
Supplement: Supplemental Material S2 [file JSLHR-64-2438-s002.pdf]

Supplemental Material S2. Effect of phonological complexity on variability.

		Variability (CoV)		
Predictors	*Std. Beta*	*Estimates*	*CI*	*p*
(Intercept)		0.18	0.15 – 0.20	**<0.001**
Group PWS:PWTF	-0.26	-0.04	-0.08 – 0.00	0.077
Word 4c:4s	-0.04	-0.00	-0.03 – 0.03	0.932
Articulator Lip:Velum.	-0.11	-0.05	-0.08 – -0.03	**<0.001**
Articulator Lip:Tongue	-0.15	-0.02	-0.05 – 0.00	0.086
Articulator Velum:Tongue	0.11	0.03	0.01 – 0.06	**0.008**
Group PWS:PWTF * Word 4c:4s	-0.38	0.02	-0.02 – 0.07	0.382
Word 4c:4s * Lip:Velum	-0.01	-0.02	-0.05 – 0.02	0.275
Word 4c:4s * Lip:Tongue	0.00	-0.00	-0.04 – 0.03	0.894
Word 4c:4s * Velum:Tongue	0.00	0.02	-0.02 – 0.05	0.337
Group PWS:PWTF * Lip:Velum	-0.02	0.00	-0.04 – 0.04	0.981
Group PWS:PWTF * Lip:Tongue	-0.01	-0.01	-0.05 – 0.03	0.714
Group PWS:PWTF * word 4c:4s * Lip:Velum	-0.02	-0.01	-0.06 – 0.04	0.706
Group PWS:PWTF * word 4c:4s * Lip:Tongue	0.12	-0.01	-0.06 – 0.05	0.822
Group PWS:PWTF * Word 4c:4s * Velum:Tongue	0.04	0.00	-0.05 – 0.06	0.878
**Random Effects**				
Marginal R^2		0.210		
Conditional R^2		0.587		
$N_{participant}$		47		
Observations		273		
R formula = variability ~ group * word * articulator + (1 + word | p_code), REML= TRUE, contrasts = contra.sum				
