# Supplementary material for: Speech Movement Variability in People Who Stutter: A Vocal Tract Magnetic Resonance Imaging Study
Source: J Speech Lang Hear Res. 2021 Jun 22;64(7):2438–52. doi: 10.1044/2021_JSLHR-20-00507 (PMC8323486; doi:10.1044/2021_JSLHR-20-00507)
Supplement: Supplemental Material S3 [file JSLHR-64-2438-s003.pdf]

Supplemental Material S3. Effect of word length on duration

		Mean Duration (frames)		
Predictors	*Std. Beta*	*Estimates*	*CI*	*p*
(Intercept)		6.87	5.46 – 8.29	<0.001
Group PWS:PWTF	0.03	0.82	-1.37 – 3.00	0.465
Word 1:2	0.63	15.75	14.83 – 16.67	<0.001
Word 1:3	1.12	28.57	27.62 – 29.52	<0.001
Word 2:3	0.5	12.82	11.87 – 13.76	<0.001
Group PWS:PWTF * Word 1:2	-0.05	-1.74	-3.16 – -0.31	0.017
Group PWS:PWTF * Word 1:3	-0.12	-4.10	-5.54 – -2.66	<0.001
Group PWS:PWTF * Word 2:3	-0.07	-2.36	-3.81 – -0.92	0.001
**Random Effects**				
Marginal R^2		0.857		
Conditional R^2		0.938		
$N_{participant}$		48		
Observations		411		

R formula = mean_duration ~ group * word + (1 | p_code), REML = TRUE, contrasts = contr.sum
