# Supplementary material for: Speech Movement Variability in People Who Stutter: A Vocal Tract Magnetic Resonance Imaging Study
Source: J Speech Lang Hear Res. 2021 Jun 22;64(7):2438–52. doi: 10.1044/2021_JSLHR-20-00507 (PMC8323486; doi:10.1044/2021_JSLHR-20-00507)
Supplement: Supplemental Material S4 [file JSLHR-64-2438-s004.pdf]

Supplemental Material S4. Effect of phonological complexity on duration

		Mean Duration (frames)		
Predictors	*Std. Beta*	*Estimates*	*CI*	*p*
(Intercept)		49.33	46.55 – 52.10	<0.001
group PWS:PWTF	-0.26	-6.21	-10.47 – -1.96	0.004
Word 4c:4s	-0.77	-17.82	-18.69 – -16.95	<0.001
Group PWS:PWTF * word 4c:4s	0.09	2.51	1.15 – 3.87	<0.001
**Random Effects**				
Marginal R^2		0.568 /		
Conditional R^2		0.944		
N_{p_code}		47		
Observations		273		

R formula = mean_duration ~ group * word + (1 | p_code), REML = TRUE, contrasts = contr.sum
